# Methylphenidate modulates the ınflammatory response and PI3K signaling in macrophages

**DOI:** 10.1007/s00210-026-05027-z

**Published:** 2026-01-22

**Authors:** Semanur Ercan, Batuhan Yurtseven, Ömer Mete Başkan, Ece Aydın, Melek Ebrar Emer, Esra Aydemir, Furkan Ayaz

**Affiliations:** 1 Department of Molecular Biology and Genetics, Faculty of Engineering and Natural Sciences, Biruni University, Istanbul, 34010 Turkey; 2 Department of Molecular Biology and Genetics, Faculty of Engineering and Natural Sciences, Istinye University, Istanbul, 34003 Turkey

**Keywords:** Cytokine, Inflammation, MPH, PI3K pathway, Macrophage cells, LPS

## Abstract

This study explored the potential immunomodulatory role of methylphenidate (MPH), specifically focusing on its modulation of the inflammatory response and the phosphoinositide 3-kinase (PI3K) signaling pathway in J774.2 murine macrophages. Cells were treated with increasing concentrations of MPH (1, 5, and 10 μg/mL) in the presence or absence of lipopolysaccharide (LPS). Cytokine concentrations (TNF-α, IL-6, IL-12p40, and GM-CSF) were quantified using ELISA, and intracellular activation of the PI3K signaling pathway was assessed via flow cytometry. It was demonstrated that MPH strongly suppressed the inflammatory cytokines TNF-α, IL-6, and IL-12p40 in a dose-dependent manner (*p* < 0.001), with significant suppression of GM-CSF evident at higher doses. Furthermore, flow cytometric analysis revealed that PI3K signaling was modulated in the presence of LPS, indicating a complex regulatory mechanism. Collectively, these findings suggest that MPH holds therapeutic potential for neuroinflammatory disorders through the dual action of suppressing inflammatory cytokines and modulating PI3K signaling pathways.

## Introduction

Methylphenidate (MPH) is the primary psychostimulant prescribed for the management of attention-deficit/hyperactivity disorder (ADHD) and narcolepsy. Its pharmacological mechanism is well-characterized, functioning primarily as a norepinephrine and dopamine reuptake inhibitor (NDRI), thereby increasing the synaptic concentrations of these monoamines in the central nervous system (CNS) (Silczuk et al. 2025). While the neurocognitive benefits of MPH are extensively documented, its systemic effects, particularly on the immune system, remain largely unexplored. Emerging evidence highlights a bidirectional communication axis between the CNS and the immune system, where neurotransmitters such as dopamine act not only as neuromodulators but also as potent regulators of peripheral immune responses (Channer et al. 2023; Yang et al. 2025). Given that MPH significantly alters dopaminergic tone, it is hypothesized to possess immunomodulatory properties that extend beyond its behavioral effects.

Macrophages are critical effectors of the innate immune system, capable of initiating inflammatory cascades through the secretion of cytokines such as tumor necrosis factor-alpha (TNF-α), interleukin-6 (IL-6), and interleukin-12 (IL-12) upon stimulation with pathogen-associated molecular patterns like lipopolysaccharide (LPS) (Bhol et al. 2024). Notably, macrophages express functional dopamine receptors (DRD1–DRD5), rendering them susceptible to modulation by dopaminergic agents. Recent studies suggest that dopamine signaling can influence macrophage plasticity and cytokine secretion, although the direction of this modulation—pro-inflammatory versus anti-inflammatory—often depends on the concentration and receptor subtype availability (Nolan et al. 2019; Wang et al. 2025). However, the specific impact of MPH, as a dopamine transporter blocker, on macrophage-mediated inflammation remains contradictory and under-investigated in the current literature.

Intracellularly, the phosphoinositide 3-kinase (PI3K)/Akt signaling pathway serves as a pivotal regulator of macrophage survival, metabolic reprogramming, and inflammatory cytokine production (De–Leon-Lopez et al. 2023). Dysregulation of this pathway is often implicated in chronic inflammatory conditions and neuroinflammation. While the PI3K pathway is a known target for various immunomodulatory drugs, the potential of MPH to modulate this specific signaling axis during an inflammatory challenge has not been elucidated. Understanding this mechanism is crucial, as MPH is frequently administered chronically to pediatric and adult populations where immune homeostasis is vital.

Therefore, this study aims to bridge this knowledge gap by investigating the immunomodulatory and anti-inflammatory potential of MPH on LPS-stimulated J774.2 murine macrophages. Specifically, the dose-dependent effects of MPH on the secretion of key pro-inflammatory cytokines (TNF-α, IL-6, IL-12p40, and GM-CSF) were examined, and the involvement of the PI3K signaling pathway was evaluated using flow cytometric analysis.

## Materials and methods

### Cell culture and reagents

The mammalian macrophage cell line J774.2 was obtained from the American Type Culture Collection (ATCC) and cultured in Roswell Park Memorial Institute (RPMI) 1640 medium supplemented with 10% fetal bovine serum (FBS) and 1% penicillin–streptomycin antibiotics. Cells were maintained in a humidified incubator at 37 °C with 5% CO2. Methylphenidate (MPH) (Sigma-Aldrich, USA) was dissolved in sterile distilled water to prepare a stock solution. Lipopolysaccharide (LPS) from *Escherichia coli* was used as the inflammatory stimulus.

### Cell viability assay

To determine non-cytotoxic concentrations of MPH, cell viability was assessed using the Trypan Blue exclusion method. J774.2 macrophages were seeded in 24-well plates at a density of 1 × 10^5^ cells/well and allowed to adhere overnight. Cells were then treated with increasing concentrations of MPH in the presence or absence of LPS (1 μg/mL) for 24 h. Following incubation, cells were harvested, stained with Trypan Blue, and viable cells were counted using a hemocytometer. Cell viability was expressed as a percentage relative to the control group.

### Experimental design and drug treatment

For cytokine and signaling assays, macrophages were seeded in 24-well plates and incubated overnight to ensure attachment. The cells were divided into four experimental groups: (i) negative control (untreated cells), (ii) LPS control (cells stimulated with 1 μg/mL LPS), (iii) MPH alone (cells treated with 1, 5, or 10 μg/mL MPH), and (iv) MPH + LPS (cells co-treated with 1 μg/mL LPS and varying concentrations of MPH). All treatments were performed in triplicate.

### Measurement of cytokine levels (ELISA)

Levels of TNF-α, IL-6, IL-12p40, and GM-CSF in culture supernatants were quantified using commercial sandwich ELISA kits (BD Biosciences) according to the manufacturer’s instructions. Briefly, 96-well plates were coated with specific capture antibodies and incubated overnight at 4 °C. After blocking, supernatants were added and incubated for 2 h at room temperature. Plates were then washed and incubated with biotinylated detection antibodies and streptavidin-HRP conjugate. The reaction was developed using TMB substrate and stopped with 1 M sulfuric acid. Absorbance was measured at 450 nm using a microplate reader. Cytokine concentrations were calculated from standard curves (Önal et al. 2023; Yurtseven et al. 2025).

### Flow cytometric analysis of PI3K activation

Intracellular activation of the PI3K signaling pathway was assessed using flow cytometry. Following 24-h treatment with MPH (10 μg/mL) in the presence or absence of LPS, cells were harvested, fixed, and permeabilized using the Cyto-Fast™ Fix/Perm Buffer Set. Cells were then stained with specific anti-PI3K antibodies for 30 min in the dark. After washing, cells were resuspended in PBS containing 1% FBS and analyzed using a flow cytometer. Data were expressed as the percentage of PI3K-positive cells within the gated population (Atalay et al. 2025).

### Statistical analysis data

Data are presented as mean ± standard error of the mean (SEM) of at least three independent experiments. Given the experimental design focusing on pre-planned independent comparisons between specific treatment groups (e.g., LPS vs. MPH + LPS) rather than all-against-all comparisons, statistical significance was determined using the independent (unpaired) Student’s *t*-test. A *p*-value of < 0.05 was considered statistically significant. All analyses were performed using GraphPad Prism software.

## Results

### MPH was used at non-cytotoxic concentrations

**Fig. 1:**
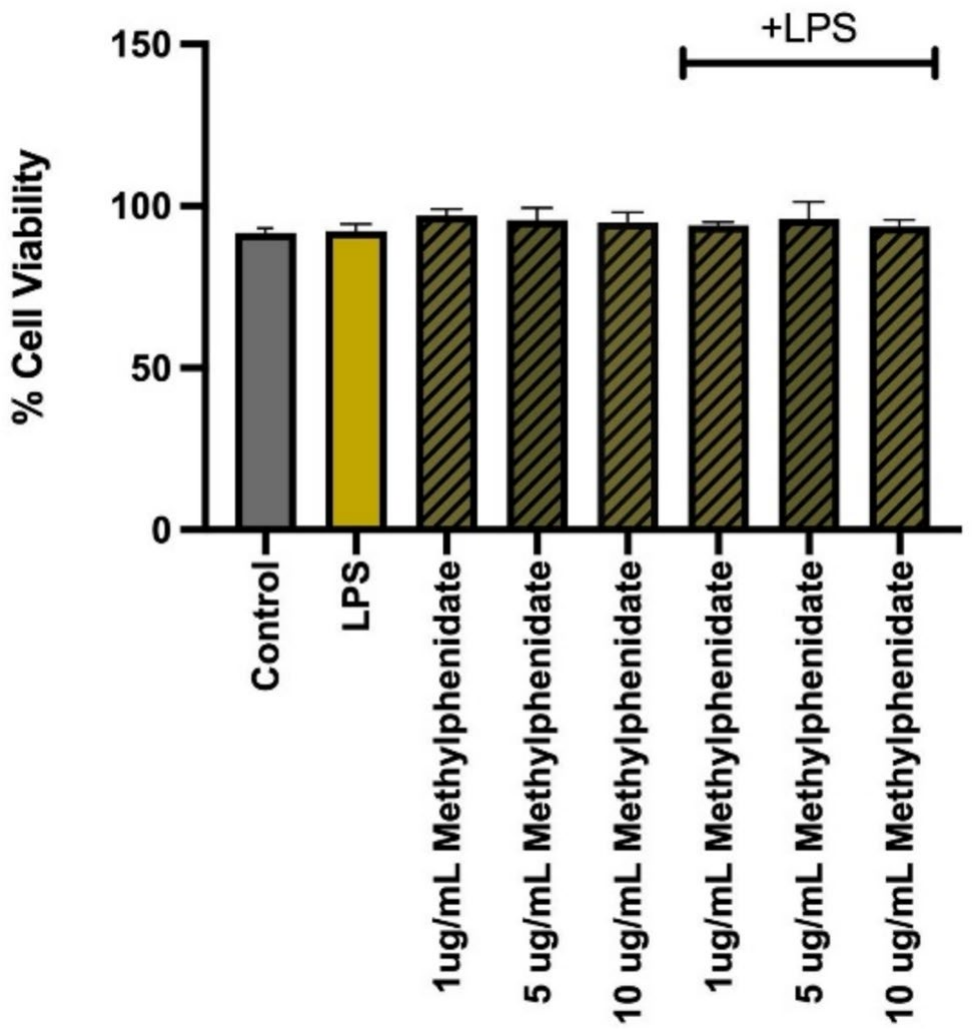
The cell viability results upon incubation with methylphenidate

To ensure that the observed immunomodulatory effects were not attributed to cytotoxicity, cell viability was assessed using the Trypan Blue exclusion assay. As demonstrated in Fig. 1, exposure of J774.2 macrophages to MPH at concentrations ranging from 1 to 20 μg/mL for 24 h resulted in no significant reduction in cell viability compared to the control group (> 95% viability). Consequently, concentrations of 1, 5, and 10 μg/mL were established as non-toxic and selected for subsequent efficacy experiments.

### Suppression of pro-inflammatory cytokine production by MPH

**Fig. 2:**
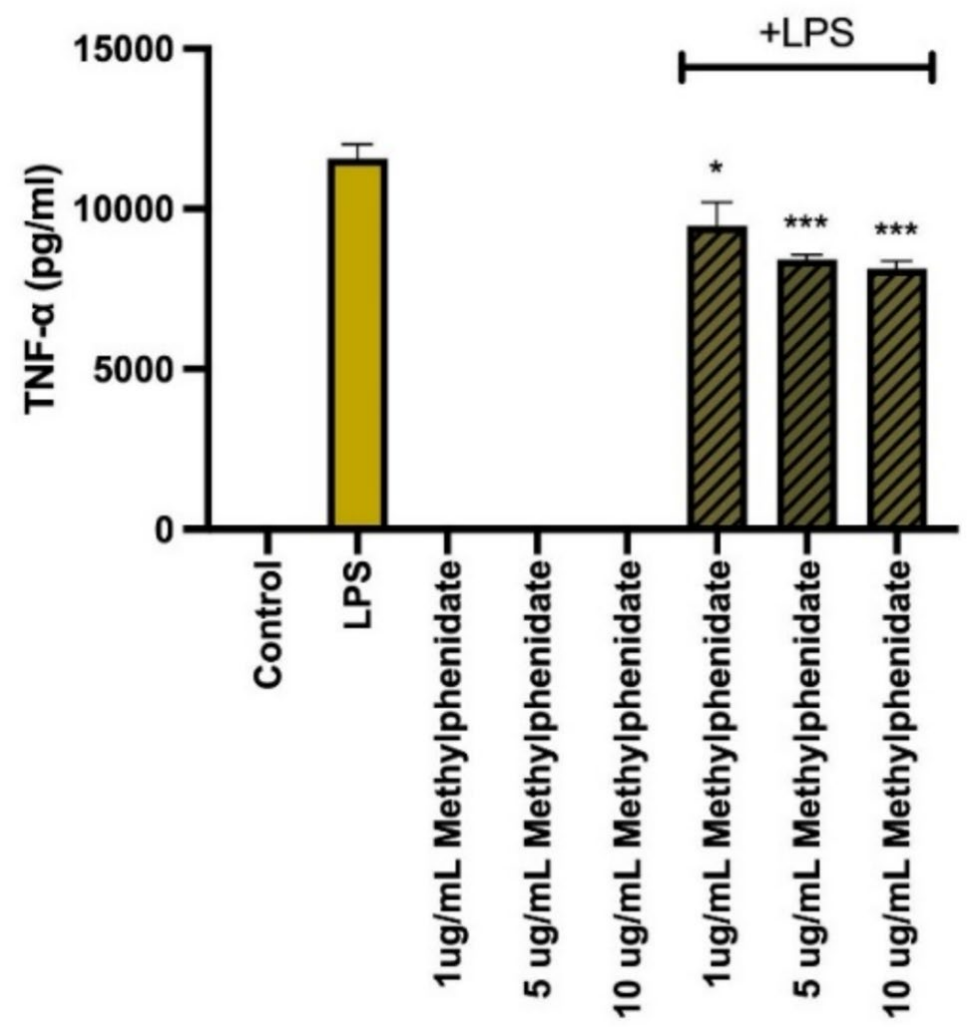
Methylphenidate (MPH) suppresses LPS-induced TNF-α production in a dose-dependent manner. As the drug concentration is increased, the amount of cytokine produced decreases (relative to control). Data are expressed as mean ± SEM (*n* = 3). *p < 0.001, **p < 0.0005, and ***p < 0.0001 indicate significant differences compared to the LPS-stimulated control group

**Fig. 3:**
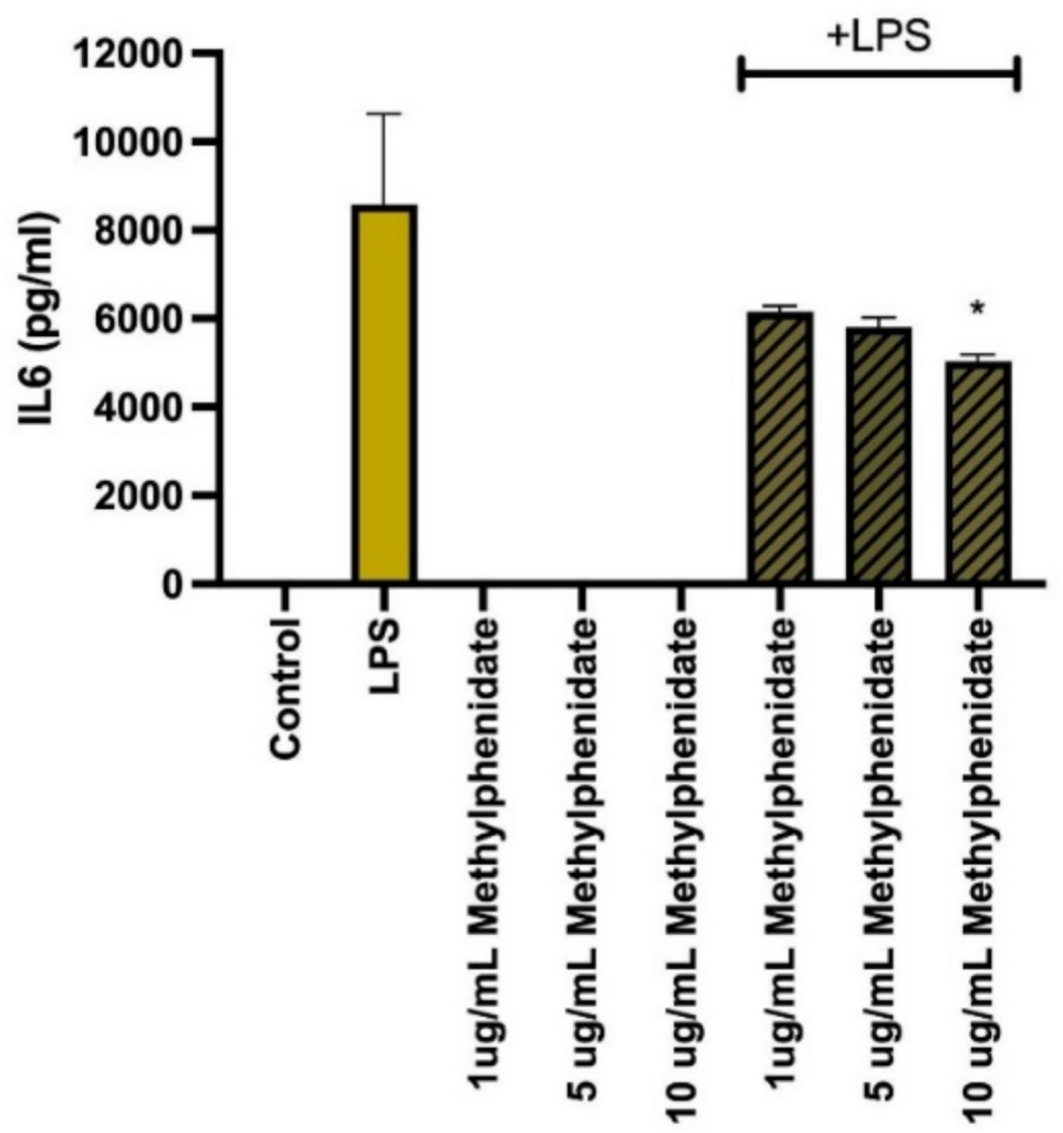
Methylphenidate (MPH) suppresses LPS-induced IL-6 production in a dose-dependent manner. As the drug concentration is increased, the amount of cytokine produced decreases (relative to control). Data are expressed as mean ± SEM (*n* = 3). *p < 0.001, **p < 0.0005, and ***p < 0.0001 indicate significant differences compared to the LPS-stimulated control group

**Fig. 4:**
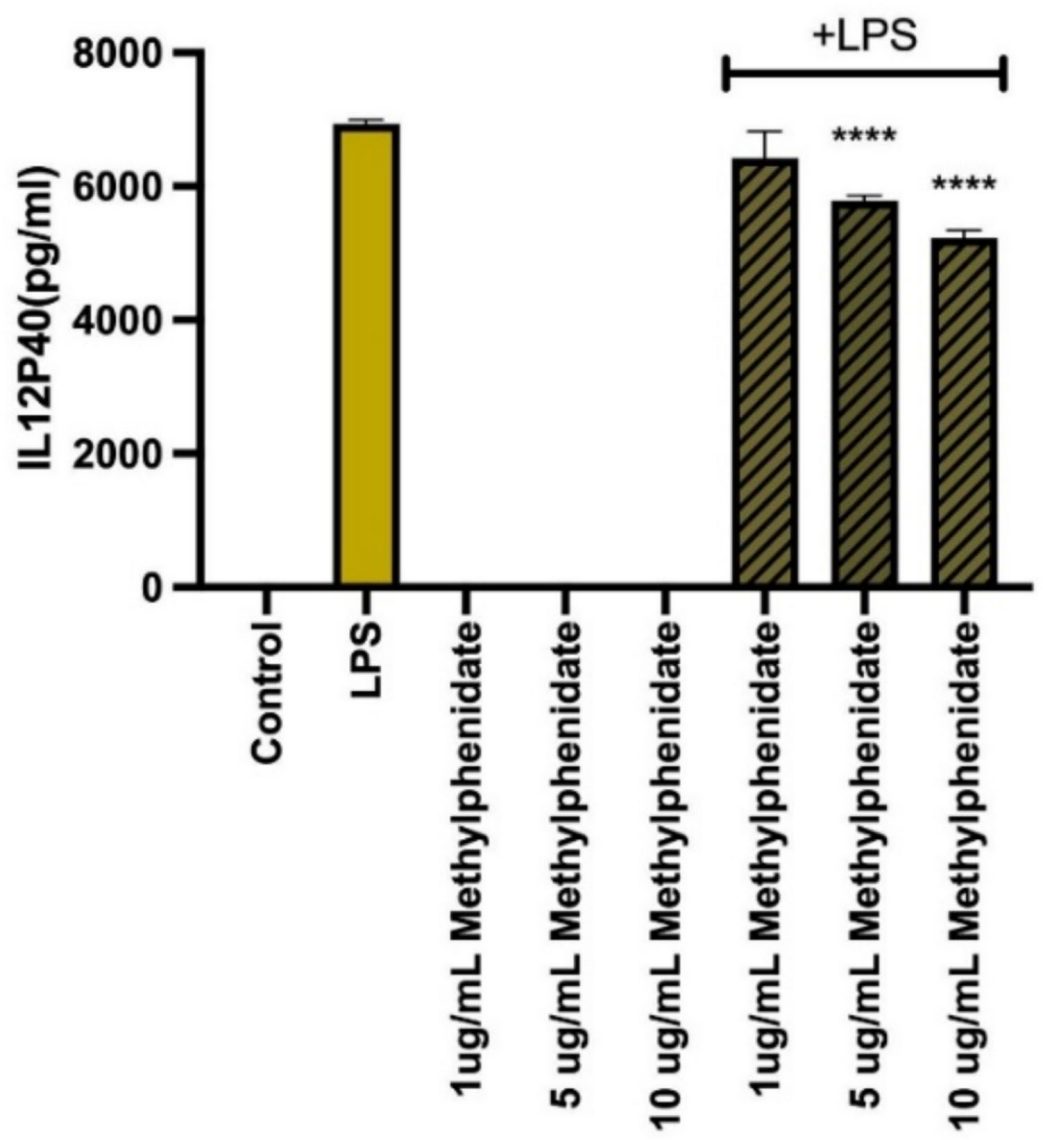
Methylphenidate (MPH) suppresses LPS-induced IL-12p40 production in a dose-dependent manner. As the drug concentration is increased, the amount of cytokine produced decreases constantly (relative to control). Data are expressed as mean ± SEM (*n* = 3). *p < 0.001, **p < 0.0005, and ***p < 0.0001 indicate significant differences compared to the LPS-stimulated control group

**Fig. 5:**
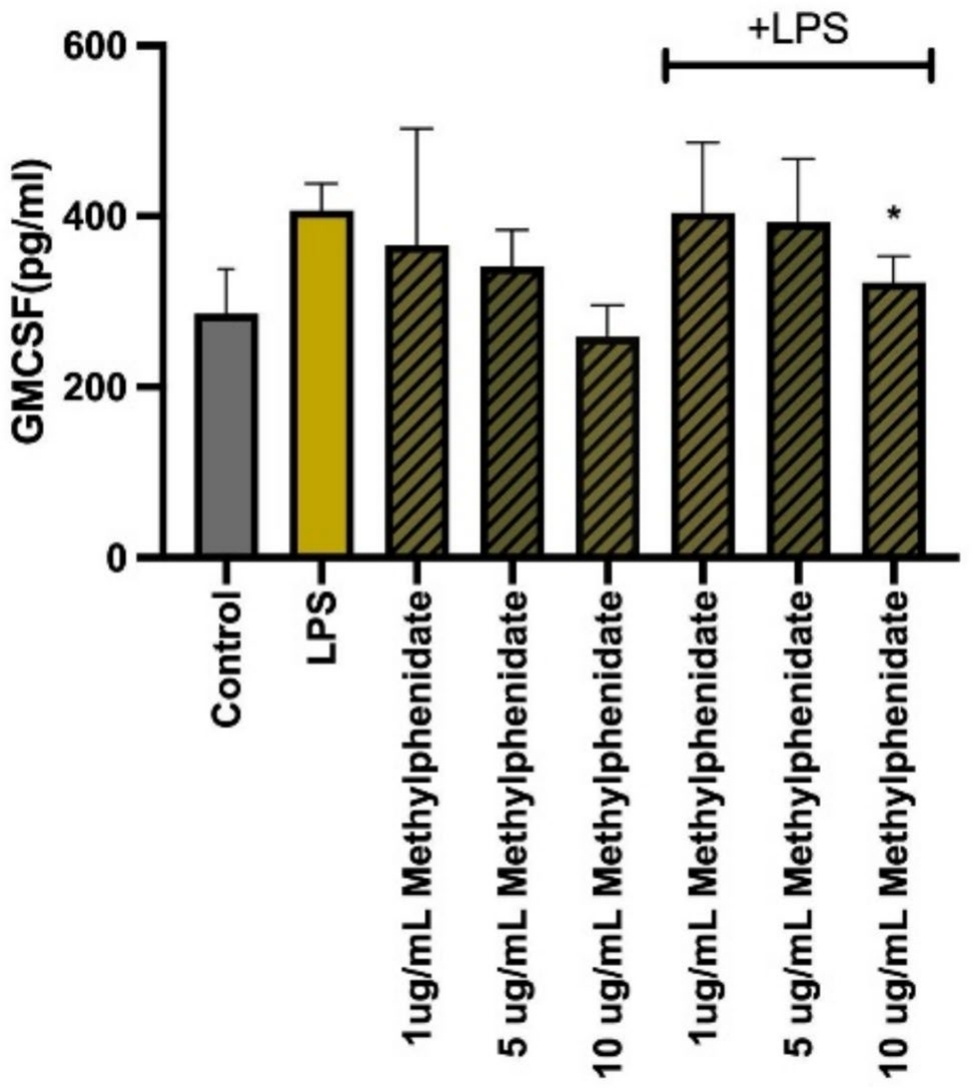
Methylphenidate (MPH) suppresses LPS-induced GMCSF production in a dose-dependent manner. The anti-inflammatory effect observed with other cytokines was not observed with GM–CSF cytokine. Data are expressed as mean ± SEM (*n* = 3). *p < 0.001, **p < 0.0005, and ***p < 0.0001 indicate significant differences compared to the LPS-stimulated control group

The modulatory effect of MPH on the secretion of key pro-inflammatory cytokines was evaluated in LPS-stimulated macrophages. As expected, LPS stimulation significantly upregulated the production of TNF-α, IL-6, IL-12p40, and GM-CSF compared to untreated controls. However, a generalized and profound suppressive effect was observed upon co-treatment with MPH. Specifically, MPH treatment resulted in a statistically significant, dose-dependent reduction in the levels of TNF-α (Fig. 2), IL-6 (Fig. 3), and IL-12p40 (Fig. 4) across all tested concentrations (*p* < 0.001). Additionally, GM-CSF secretion was markedly downregulated, particularly at higher MPH concentrations (Fig. 5) (*p* < 0.01). Importantly, it was confirmed that MPH alone (without LPS) did not induce any significant cytokine secretion, indicating the absence of intrinsic immunostimulatory activity in resting cells.

### MPH modulates LPS-induced PI3K activation in macrophages

**Fig. 6:**
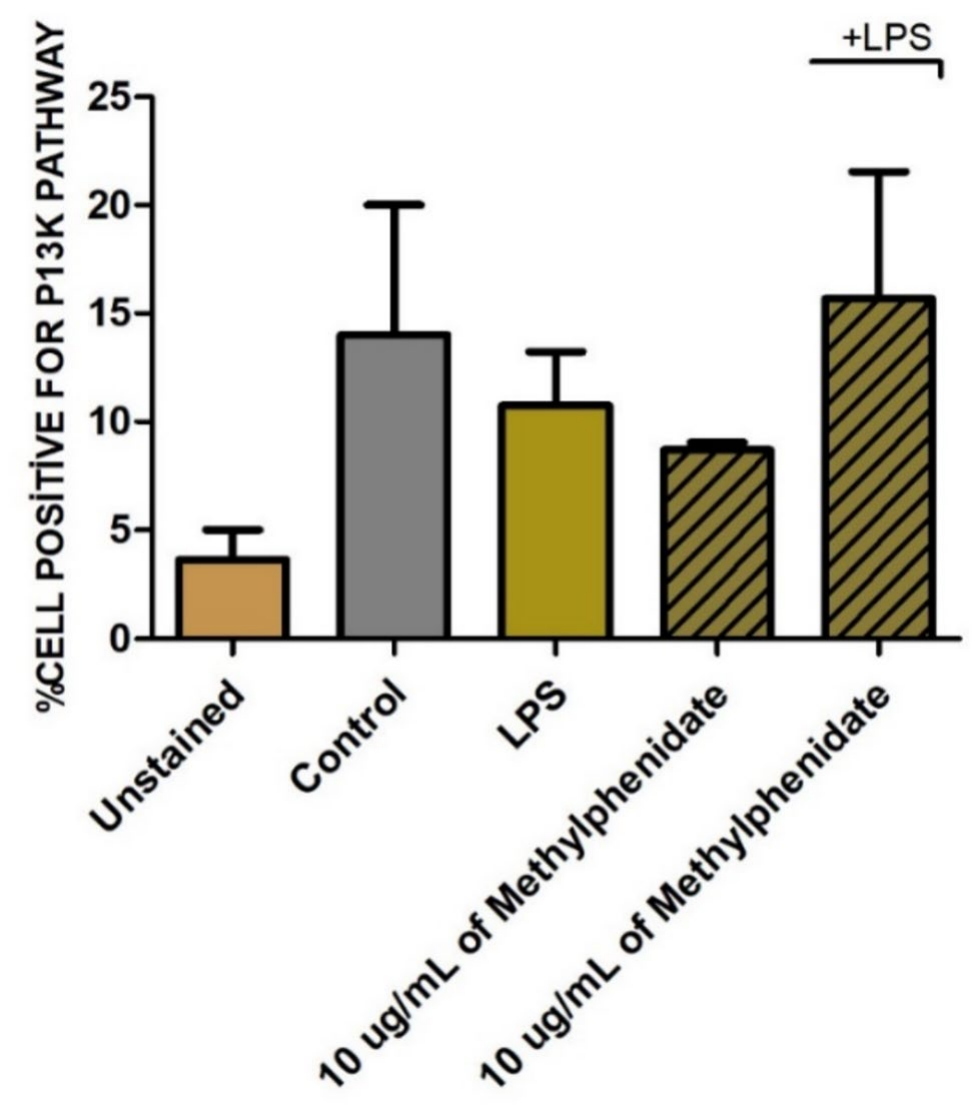
Quantification of PI3K pathway activation. The percentage of PI3K-positive cells was analyzed across experimental groups. Data are presented as mean ± SEM

**Fig. 7:**
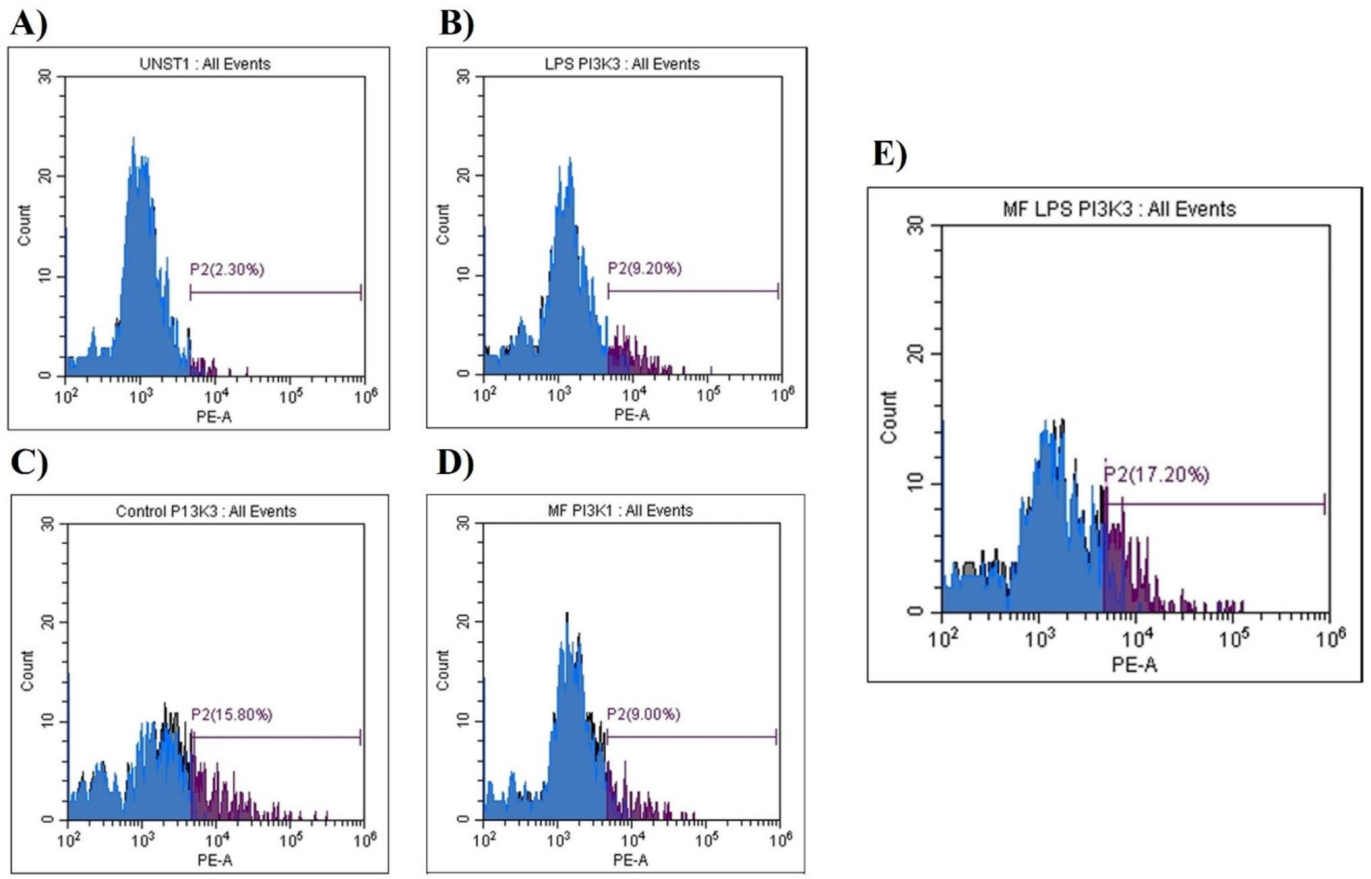
Representative flow cytometry histograms showing PI3K levels in macrophages treated with methylphenidate and LPS (P2 represents the percentage of cells within the gated population exhibiting PI3K signal pathways). **A** Unstained, **B** LPS PI3K, **C** control PI3K, **D** MPH PI3K, **E** MPH LPS PI3K

To elucidate the intracellular mechanism underlying the observed cytokine suppression, the activation of the PI3K signaling pathway was analyzed using flow cytometry. As depicted in Fig. 6, MPH treatment alone did not trigger PI3K activation compared to the negative control. In contrast, LPS stimulation led to a marked increase in the percentage of PI3K-positive cells. Upon co-treatment with MPH in the presence of LPS, a distinct modulation of this pathway was observed (Fig. 7). Although complete inhibition of PI3K was not detected, the alterations in fluorescence intensity and the percentage of activated cells suggested a complex regulatory interaction, indicating that the anti-inflammatory properties of MPH are partially mediated through the modulation of PI3K signaling dynamics.

## Discussion

The primary objective of this study was to elucidate the immunomodulatory effects of methylphenidate (MPH) on macrophage-mediated inflammation and to investigate the involvement of the PI3K signaling pathway. While MPH is widely prescribed for ADHD, its impact on peripheral immune responses has remained understudied. The findings presented here demonstrate that MPH exerts a potent, dose-dependent anti-inflammatory effect on LPS-stimulated J774.2 macrophages, characterized by the suppression of key pro-inflammatory cytokines and the complex modulation of intracellular PI3K signaling.

Consistent with the initial hypothesis regarding the interplay between dopaminergic signaling and immunity, MPH treatment significantly attenuated the production of TNF-α, IL-6, and IL-12p40. These cytokines are critical drivers of the acute inflammatory response and neuroinflammation. The suppression of IL-12p40 is particularly vital, as it bridges innate and adaptive immunity, suggesting that MPH may influence downstream T-cell responses. Furthermore, the reduction of GM-CSF at higher concentrations indicates a broader regulatory potential of MPH on myeloid cell differentiation and survival. These results align with emerging evidence suggesting that monoaminergic modulation can dampen excessive immune activation (Hall et al. 2024; Nava et al. 2025). For instance, similar anti-inflammatory properties have been reported for selective serotonin reuptake inhibitors (SSRIs) such as fluoxetine and sertraline (Önal et al. 2023; Önal et al. 2023) in macrophage models, suggesting that psychotropic agents targeting monoamine transporters may share a common immunomodulatory mechanism (Bleibel et al. 2025; Yin et al. 2024).

**Fig. 8:**
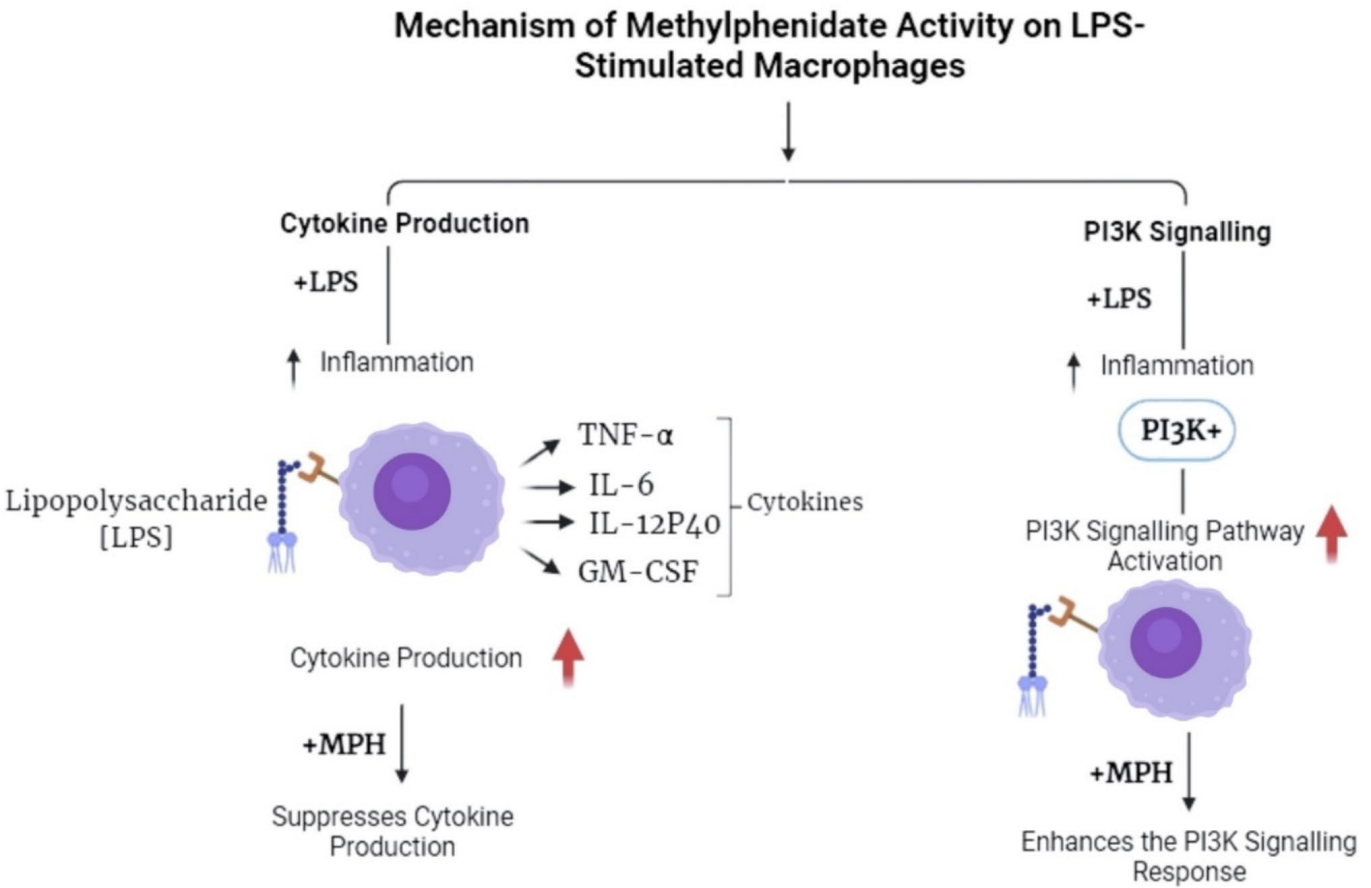
Mechanism of methylphenidate activity onLPS-stimulated macrophages

A pivotal finding of this study is the differential regulation of the PI3K signaling pathway. While LPS stimulation alone induced expected PI3K activation, co-treatment with MPH further enhanced this signal intensity, despite the concurrent suppression of pro-inflammatory cytokines. This observation presents an intriguing paradox, as the PI3K/Akt pathway is typically associated with pro-inflammatory signaling. However, recent studies indicate that PI3K activation can be dualistic (Zhu et al. 2025); it is essential for both cytokine production and the resolution of inflammation through the promotion of cell survival. It is plausible that in this context, the enhanced PI3K activation induced by MPH represents a compensatory survival signal or a negative feedback loop intended to limit cytotoxicity and restore homeostasis under inflammatory stress. Similar “uncoupling” of PI3K signaling from cytokine production has been reported in other immunopharmacological studies, suggesting that MPH may redirect macrophage signaling towards a survival or resolution phenotype rather than an effector phenotype (Fig. 8).

The clinical implications of these findings are noteworthy. Chronic neuroinflammation is increasingly recognized in the pathophysiology of various neuropsychiatric disorders. The ability of MPH to suppress macrophage-derived cytokines suggests that its therapeutic benefits in ADHD might extend beyond neurotransmitter reuptake inhibition to include neuroimmune regulation. Specifically, the reduction of TNF-α and IL-6 may be relevant for patients with comorbid inflammatory conditions, although the systemic impact in a clinical setting requires careful evaluation.

### Limitations

Several limitations of this study should be acknowledged. First, the experiments were conducted using a single murine macrophage cell line (J774.2); therefore, these findings require validation in primary human monocytes or microglia to ensure clinical translatability. Second, cell viability was assessed using the Trypan Blue exclusion method, which is less sensitive than metabolic assays such as MTT or ATP quantification. Finally, statistical analysis was conducted using the Student’s *t*-test for specific pre-planned comparisons (e.g., LPS vs. MPH + LPS) without post-hoc correction for multiple comparisons, which should be considered when interpreting the statistical strength of the findings. Future studies should employ Western blotting to identify specific downstream phosphorylation targets (e.g., Akt/mTOR) to fully map the molecular mechanism.

## Conclusion

In conclusion, our findings contribute to the growing body of evidence supporting the immunomodulatory role of pharmacological agents and highlight the potential of MPH (MPH) in regulating inflammatory responses in macrophages.

The observed suppression of TNF-α, IL-6, IL-12p40, and GM-CSF production suggests that MPH may exert anti-inflammatory effects in a dose-dependent manner. Furthermore, activating the PI3K signalling pathway in response to MPH treatment highlights its potential role in immune regulation. These findings contribute to a growing body of research on the immunomodulatory effects of pharmacological agents and suggest that MPH may have therapeutic implications in conditions associated with excessive inflammation. However, further studies are needed to elucidate this compound’s precise molecular mechanisms and potential clinical applications in immune-related disorders.

## Data Availability

The datasets generated during and/or analysed during the current study are available from the corresponding author on reasonable request.
